# Temporal Trends in Gender Affirmation Surgery Among Transgender and Non-Binary Minors

**DOI:** 10.7759/cureus.45948

**Published:** 2023-09-25

**Authors:** Bashar Hassan, Ferris Zeitouni, Mona Ascha, Renata Sanders, Fan Liang

**Affiliations:** 1 Center for Transgender and Gender Expansive Health, Johns Hopkins University, Baltimore, USA; 2 School of Medicine, Texas Tech University Health Sciences Center, Lubbock, USA

**Keywords:** transgender and non-binary, minors, gender affirmation, chest masculinization surgery, adolescents

## Abstract

Background: Over the last decade, a greater number of transgender or non-binary (TGNB) minors have been seeking gender affirmation surgery (GAS). Given the recent concerns about the potential harm of GAS in TGNB minors, we sought to determine the incidence and postoperative outcomes of GAS among TGNB minors.

Methods: We retrospectively reviewed the American College of Surgeons National Surgical Quality Improvement Program (ACS NSQIP) Pediatric database, 2018-2021, for minors aged 17 years or younger. The primary outcome was the frequency and type of GAS plotted over time. The secondary outcome was the incidence of postoperative complications within 30 days following GAS. Descriptive statistics were calculated. Linear regression was performed to assess the association between the incidence of GAS and time in years.

Results: A total of 108 TGNB minors were identified. The mean (SD) age was 16.9 (0.8) years without significant variation over time. There was a significant increase in the number of GAS per year over four years (*P*<.001). Nevertheless, only two (1.9%) patients underwent GAS below the age of 15 (13.9 and 14.5 years). Chest masculinization surgery (CMS) was the predominant procedure type among TGNB minors (n*=*102, 94.4%). There was no incidence of major complications (mortality, bleeding, sepsis, unplanned intubation) except for unplanned reoperation for hematoma evacuation (n*=*3, 2.8%). The incidence of minor complications (surgical site infection, wound dehiscence) was low (n*=*1, 0.9%), each).

Conclusions and relevance: GAS in minors, primarily in the form of CMS, has been increasing over time. CMS in minors is a safe procedure with rare complications.

## Introduction

The prevalence of transgender or non-binary (TGNB) adolescents in the United States is estimated to range from 2.7% to 9.2% [1,2]. Recent data by the Williams Institute reveals that approximately 300,000 adolescents aged 13-17 years identify as TGNB [3]. This rise in the frequency of TGNB individuals is accompanied by increasing demand for gender-affirming care (GAC) [4,5], including gender-affirming surgery (GAS) [5-7]. However, access to GAC in TGNB minors in many areas of the United States remains a significant challenge [8].

Gender dysphoria refers to the discrepancy between an individual's gender identity and their assigned gender at birth [9]. This condition has a historical precedent, with documented hormone therapy treatment and the first reported GAS dating back to the 1930s [10]. The World Health Organization defines GAC as social, psychological, behavioral, or medical interventions that validate and support an individual's gender identity [11]. GAC can involve hormone therapy, surgical intervention, or a combination of both. Common surgical procedures include chest masculinization/feminization surgery, hysterectomy, genital surgery (vaginoplasty, metoidioplasty, phalloplasty), facial feminization, voice surgery, and body contouring [12-14]. Chest masculinization surgery (CMS) is the most commonly performed GAS for both adult and adolescent TGNB individuals [4,5,15,16].

Hormone therapy may be initiated after the onset of puberty and can include pubertal blockers, testosterone, or estradiol therapy [14,17]. The positive effects of hormone therapy in minors have been well documented, with studies indicating a reduction in depression, suicidal ideation, and body dissatisfaction after initiation [18,19]. Similarly, GAS such as CMS, has been shown to decrease chest dysphoria and improve body image satisfaction and quality of life for TGNB individuals [20-22]. This is particularly important in TGNB minors who often experience pressure to conform to societal norms and difficulty “fitting in” [10]. The increased psychosocial distress can further contribute to the higher rates of depression, anxiety, and suicidal ideation TGNB minors face when compared to their cisgender counterparts [2,8,10]. Access to comprehensive care, including GAS, can help TGNB minors navigate the challenges of gender dysphoria in adolescence [20,22].

Although there is a growing body of literature on GAS and studies indicating an increase in CMS requests among minors over time, there is limited research examining surgical trends and outcomes in TGNB minors, particularly those 17 years old and younger [6,7]. Our study is the first multi-institutional analysis using the American College of Surgeons National Surgical Quality Improvement Program (ACS NSQIP) Pediatric registry to investigate the surgical trends and outcomes of TGNB minors. We hypothesize that while the frequency of GAS in TGNB minors is increasing over time, significant complications are rare in this patient population. Our aim is to better understand this unique and growing patient population and the safety of the services they seek.

## Materials and methods

Dataset

The ACS NSQIP Pediatric database was queried from 2018 to 2021 for TGNB minors who underwent GAS. Research involving the participation of minors requires careful consideration of patient confidentiality and privacy. Minors may have difficulty fully comprehending the implications of their participation. Therefore, it is important to obtain informed consent from both the minor and their legal guardian when using data containing patient identifiers. However, the ACS NSQIP Pediatric database does not involve “human subjects” and contains no identifiable private information. The database contains information on demographics, preoperative comorbidities, operative procedures, and 30-day postoperative surgical outcomes of surgical pediatric patients who were 17 years old or less. The data is collected by trained surgical clinical reviewers across hundreds of medical centers inside and outside the United States with the aim of improving surgical care [23]. Exempt approval was granted by the Johns Hopkins University Institutional Review Board to conduct this study.

Patient selection and stratification

Due to the inaccurate gender reporting in the NSQIP Pediatric database, TGNB minors were identified by selecting patients with an International Classification of Diseases (ICD)-9 or ICD-10 code indicating gender dysphoria or gender identity disorder as the reason for their surgery. Patients were stratified based on the type of surgery they received (e.g., chest masculinization, breast augmentation, hysterectomy, vaginoplasty, phalloplasty, facial feminization, vocal cord surgery) using current procedural terminology (CPT) codes. ICD-9, ICD-10, and CPT codes used for patient selection and stratification are shown in Table 1.

**Table 1:** ICD-9, ICD-10, and CPT codes used for patient selection and stratification ICD: International Classification of Diseases, CPT: Current Procedural Terminology

ICD-9 codes for gender dysphoria	302.50, 302.51, 302.52, 302.53, 302.6, 302.85
ICD-10 codes for gender dysphoria	F64.0, F64.1, F64.2, F64.8, F64.9
CPT codes for chest masculinization surgery	15200, 19318
CPT codes for hysterectomy	58150, 58542
CPT codes for vaginoplasty	57110
CPT codes for head and neck surgery	30410

Outcomes and covariates

Our primary outcomes were patient demographics and surgical characteristics over time. Demographic variables assessed included patient age, body mass index (BMI), and race/ethnicity. Surgical characteristics evaluated included GAS frequency and type, total operating time, and length of total hospital stay. Our secondary outcome was the incidence of postoperative complications within 30 days following GAS. Postoperative outcomes assessed included mortality, bleeding requiring transfusion, sepsis, unplanned reoperation, unplanned readmission, unplanned intubation, unplanned extubation, surgical site infection, and wound dehiscence. The definitions of these variables are included in the User Guide for the ACS NSQIP Participant Use Data File (PUF) [24].

Statistical analysis

Mean and standard deviation (SD) were used to present normally distributed numerical data, while median and interquartile range (IQR) were used to present non-normally distributed numerical data. Descriptive statistics were calculated. Linear regression was performed to assess the association between the incidence of GAS and time (per year). Statistical analysis was performed using IBM SPSS Statistics for Windows, Version 28.0 (Released 2021; IBM Corp, Armonk, New York, United States). A P-value <.05 was considered significant.

## Results

Demographics and surgical characteristics

**Figure 1:**
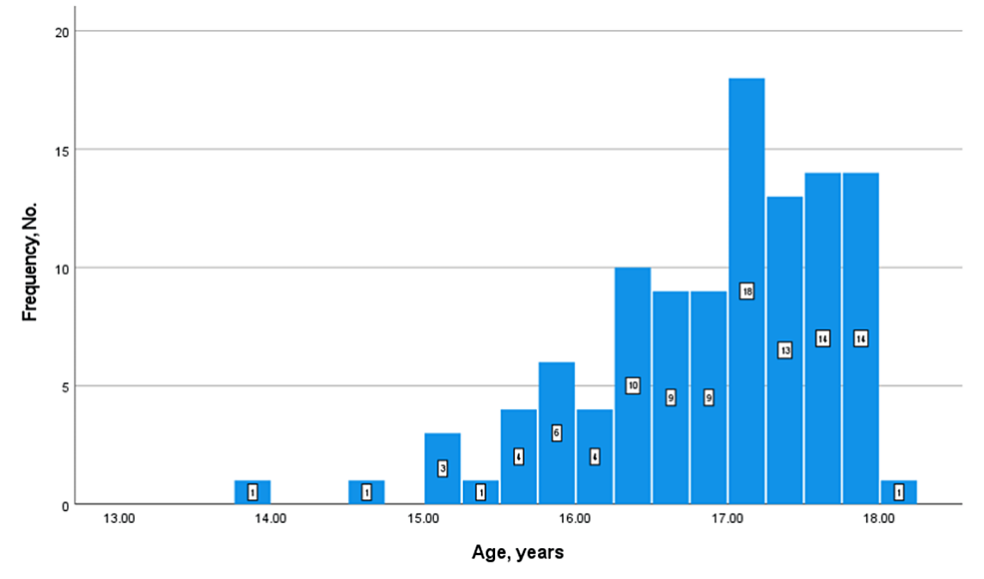
Histogram showing the age distribution of our study population. No.: number

Of 522,605 pediatric patients included in NSQIP Pediatric from 2018 to 2021, 108 (0.02%) were TGNB minors who underwent GAS and were included in our study. Table 2 displays the patient demographics and surgical characteristics of our study population. The mean (SD) age was 16.9 (0.8) years, with the youngest patient being 13.9 years old. The age distribution of our study population is depicted in Figure 1. It is worth noting that only two (1.9%) patients were under the age of 15 (13.9 and 14.5 years).

The study population had a mean (SD) BMI of 27.8 (8.3) kg/m^2^, with 79 (73.1%) patients being non-obese (<30 kg/m^2^), 22 (20.4%) being obese (30-39.9 kg/m^2^), three (2.8%) being morbidly obese (40-49.9 kg/m^2^), and four (3.7%) being super morbidly obese (BMI ≥50 kg/m^2^). The mean (SD) total operating time was 142.9 (47.5) minutes. Non-Hispanic White patients accounted for the majority of the study population (n=52, 48.1%), while non-Hispanic Black patients were the least represented (n=1, 0.9%). The most common type of GAS was CMS (n=102, 94.4%) (Table 2). Length of total hospital stay ranged between zero and one day. The majority of chest masculinization surgeries were done on an outpatient basis (n=58/102, 56.9%). All genital surgeries were done on an inpatient basis.

**Table 2:** Demographics and surgical characteristics of the study population. SD: standard deviation, IQR: interquartile range, BMI: body mass index

Demographics/Surgical Characteristics		Value
Age, mean (SD), years		16.9 (0.8)
Age, median (IQR), years		17.1 (16.4-17.5)
Age, absolute range, years		13.9-18.0
BMI, mean (SD), kg/m^2^		27.8 (8.3)
BMI categories, No. (%)	<30	79 (73.1)
	30-40	22 (20.4)
	40-50	3 (2.8)
	>50	4 (3.7)
Total operating time, mean (SD), minutes		142.9 (47.5)
Length of total hospital stay, median (IQR), days		0 (0-1)
Race/ethnicity, No. (%)	Hispanic	10 (9.3)
	Non-Hispanic White	52 (48.1)
	Non-Hispanic Black	1 (0.9)
	Non-Hispanic, unknown race	13 (12.0)
	Unknown race and ethnicity	32 (29.6)
Gender affirmation surgery, No. (%)	Chest masculinization surgery	102 (94.4)
	Hysterectomy	4 (3.7)
	Vaginectomy	1 (0.9)
	Facial feminization	1 (0.9)

Surgical trends

**Figure 2:**
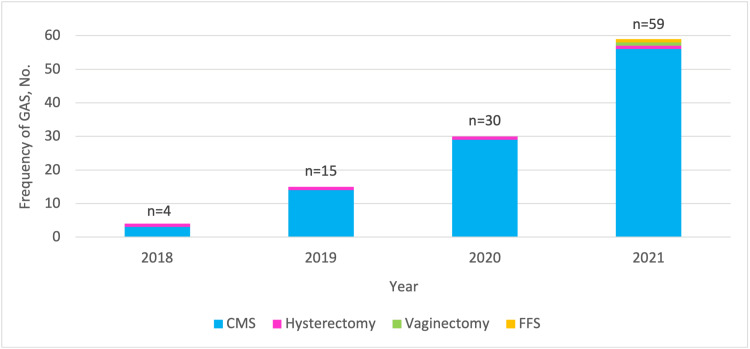
Frequency (number) of the different types of gender affirmation surgeries undergone by transgender and non-binary minors over time (years). GAS: gender affirmation surgery, No.: number, CMS: chest masculinization surgery, FFS: facial feminization surgery n = total number of GAS per year

Between 2018 and 2021, the annual number of GAS increased by 1,375% (four in 2018 and 59 in 2021). Over this four-year period, the mean (SD) age at surgery did not significantly vary and ranged from 16.9 (0.5) to 17.0 (1.3) years. Figure 2 shows the frequency of the different types of GAS undergone by TGNB minors over time. On linear regression, there was a significant increase in the number of GAS per year over four years (P<.001). The majority of patients underwent GAS in 2021 (n=59, 54.6%).

CMS was the most common GAS each year, from 2018 to 2021. Only one (0.9%) patient underwent hysterectomy each year (total n=4, 3.7%). Vaginectomy and facial feminization surgery were performed in one (0.9%) patient in 2021, each (Figure 2).

Postoperative outcomes

**Figure 3:**
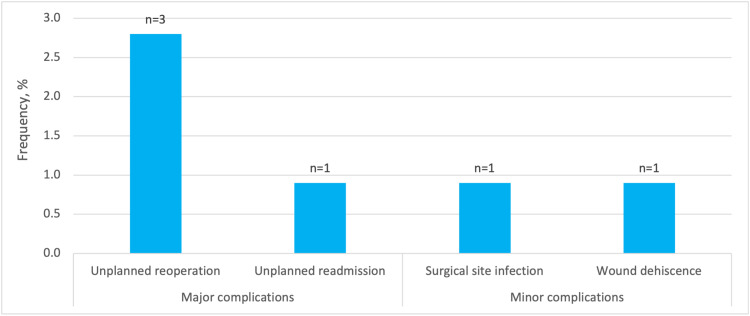
Frequency (%) of 30-day postoperative complications. The numbers at the top of the columns refer to the number of each complication

Figure 3 shows the frequency of postoperative complications within 30 days of GAS in our study population. There were no major complications in the form of mortality, bleeding requiring transfusion, sepsis, unplanned intubation, and unplanned extubation. There was a low incidence of other major complications, namely, unplanned reoperation for hematoma evacuation (n=3, 2.8%) and unplanned readmission in one (0.9%) of these patients. Among minor complications assessed, there was a low incidence of SSI (n=1, 0.9%) and wound dehiscence (n=1, 0.9%) (Figure 3).

## Discussion

Our study is the first multi-institutional study based on a national registry to describe temporal trends and postoperative outcomes in TGNB minors. Despite an increase in GAS over time, few postoperative complications occurred within 30 days of surgery, reflecting the safety of CMS in this population. Our findings provide further evidence showing the increasing demand for GAS among minors and signify the need for more readily available and accessible resources for this patient population.

Recent studies have shown an increase in the number of minors requesting GAC and a corresponding increase in those requesting GAS [5-7]. This finding mirrors trends previously described in the adult population [15,16]. CMS has been reported to be the most commonly performed GAS in both TGNB adults and minors [4-7,15]. Consistent with previous findings, CMS was the most common GAS in our study population and has been shown to significantly decrease chest dysphoria and increase body image satisfaction [20,21].

We observed more than a 1000% increase in the frequency of GAS, specifically CMS, between 2018 and 2021. These findings support previous metrics reported by Das et al. who reported a 389% increase in gender-affirming chest surgeries captured within the Nationwide Ambulatory Surgery Sample (NASS) database between 2016 and 2019 [7]. We postulate that the growth in surgical volume is non-linear; our study sample captures two additional years after the Das study, and thereby reports a three-fold higher growth than what is described by Das et al.

This increase in CMS frequency over time can potentially be explained by several factors. First, there has been a recent increase in the frequency of minors identifying as TGNB and seeking GAC, including GAS [4]. Second, healthcare providers and medical centers are increasingly recognizing the importance of transgender and gender-diverse healthcare, and access to care has grown. Third, the increased societal acceptance of CMS in minors might lead to more individuals feeling comfortable coming out and seeking medical intervention to align their bodies with their gender identities. Lastly, compared to genital GAS in minors, CMS has more reversible outcomes and is often more tolerated by both the public and medical providers in that it does not impact future fertility. In our study population, only one minor underwent hysterectomy each year from 2018 to 2021.

Despite the observed rise in CMS frequency over the years, a significant proportion of TGNB individuals do not yet have proper access to GAC due to suboptimal insurance coverage for TGNB youth. Although current international practice guidelines do not specify a minimum age after which CMS is permitted [25], the vast majority of insurance companies in the United States consider 18 years to be the minimum age for CMS [22,26]. In an analysis by Dowshen et al. of online health insurance plan indications, less than half of the plans indicated coverage for GAC in TGNB youth [27]. Similarly, 50% of respondents to the National Transgender Discrimination Survey reported denial of insurance coverage for their desired GAS [28]. This has led the majority of TGNB individuals seeking CMS to resort to self-pay options for surgical coverage [22]. Indeed, TGNB adolescents who have not undergone CMS report financial constraints as the most significant barrier to surgery [22]. This is compounded by United States legislative efforts to prohibit GAC for minors, purely based on age, despite conflicting recommendations from the World Professional Association for Transgender Health and the American Academy of Pediatrics [25,29]. The aforementioned information highlights the poor access of TGNB youth to GAC and GAS, leading to negative health outcomes and potential suicidality.

In our study population, the increase in GAS frequency was not accompanied by a significant change in the mean age of patients at surgery. A prior study by Handler et al. reported a trend toward younger ages of patients referred for GAS [4]. In their study, nearly 10% of patients referred to a specialty clinic in Northern California were under the age of eight, and nearly 10% of patients aged 9-13 years were referred for surgery [4]. Das et al. reported that the median (range) age of patients who underwent gender-affirming chest reconstruction in their study was 16 (12-17) years [7]. Our study population was relatively older than both populations analyzed in the two aforementioned studies, with a median (range) age of 17.1 (13.9-17.9) years. Only two patients were younger than 15 years (13.9 and 14.5), both of whom underwent CMS. Although there was a slight decrease in the minimum age at surgery over the four-year period analyzed, the mean age remained constant over time.

Postoperative complications in our study cohort were minimal. Unplanned reoperation for hematoma evacuation was the most common postoperative complication, occurring only in three patients. Minor complications e.g., surgical site infection and wound dehiscence, also occurred at very low rates (one patient each). These postoperative complication rates are comparable to findings in other studies involving adults and minors, and add to the growing body of literature demonstrating a good safety profile for CMS [6,21,30]. Given the minimal risk of complications following CMS in minors, the increasing demand for CMS, and the detrimental consequences of delayed/prohibited access to proper surgical care, we recommend implementing initiatives and healthcare policies targeting barriers to care including age restrictions, insurance coverage, among others, to help expand surgical access and meet the increasing demands of this population.

This study should be reviewed in light of a few limitations. The small sample size limits the generalizability of the results. This is partly explained by the inability of the NSQIP Pediatric database to capture the majority of the ambulatory surgical centers. Additionally, the NSQIP Pediatric database only provides postoperative follow-up data within 30 days of surgery, limiting our ability to capture long-term complications. However, most postoperative complications following CMS are likely to arise within the 30-day window captured by the NSQIP Pediatric database. This also signifies the need for larger studies with long-term follow-up after GAC in minors. Additionally, the NSQIP data relies on the accurate diagnosis and reporting of the participating institution. Finally, a significant limitation of the NSQIP Pediatric database is the lack of gender identity demographic information. To mitigate this limitation, we used ICD-9 and ICD-10 codes of gender dysphoria and gender identity disorder for proper patient selection. However, we were unable to study different gender identities over time due to the unreliability of gender reporting in the NSQIP Pediatric database. This signifies the importance of accurate gender identity reporting in national databases for proper representation and improved care of the growing TGNB adults and minors.

## Conclusions

As reflected in expansion of GAC, there has been a rise in CMS among minors, with very few minors undergoing CMS below the age of 15 years. CMS is a safe procedure in minors with rare complications. Lifting age restrictions, expanding insurance coverage, and overturning legislation prohibiting GAS in minors are important actions that can increase access to care. Expanded and more accessible resources are also essential to address the surgical needs of TGNB youth, including a further capacity to develop national data collection systems to better understand, support, and optimize the care of this population. Future studies with larger sample sizes will allow for better generalizability. Additionally, future studies with long-term follow-up are essential to capture the long-term postoperative outcomes of GAS in minors.
